# Cardiac Disease Classification Using Two-Dimensional Thickness and Few-Shot Learning Based on Magnetic Resonance Imaging Image Segmentation

**DOI:** 10.3390/jimaging8070194

**Published:** 2022-07-11

**Authors:** Adi Wibowo, Pandji Triadyaksa, Aris Sugiharto, Eko Adi Sarwoko, Fajar Agung Nugroho, Hideo Arai, Masateru Kawakubo

**Affiliations:** 1 Department of Informatics, Tembalang FSM Campus, Universitas Diponegoro, Semarang 50275, Indonesia; arissugiharto@lecturer.undip.ac.id (A.S.); ekoadisarwoko@lecturer.undip.ac.id (E.A.S.); fajar@lecturer.undip.ac.id (F.A.N.); 2 Department of Physics, Tembalang FSM Campus, Universitas Diponegoro, Semarang 50275, Indonesia; p.triadyaksa@fisika.fsm.undip.ac.id; 3 Fukuokaken Saiseikai Futsukaichi Hospital, Fukuoka 818-8516, Japan; fukuoka.hideo@gmail.com; 4 Department of Health Sciences, Faculty of Medical Sciences, Kyushu University, Fukuoka 812-8582, Japan; kawakubo.masateru.968@m.kyushu-u.ac.jp

**Keywords:** cardiac MRI, segmentation, few-shot learning, adaptive subspace classification, deep learning

## Abstract

Cardiac cine magnetic resonance imaging (MRI) is a widely used technique for the noninvasive assessment of cardiac functions. Deep neural networks have achieved considerable progress in overcoming various challenges in cine MRI analysis. However, deep learning models cannot be used for classification because limited cine MRI data are available. To overcome this problem, features from cine image settings are derived by handcrafting and addition of other clinical features to the classical machine learning approach for ensuring the model fits the MRI device settings and image parameters required in the analysis. In this study, a novel method was proposed for classifying heart disease (cardiomyopathy patient groups) using only segmented output maps. In the encoder–decoder network, the fully convolutional EfficientNetB5-UNet was modified to perform the semantic segmentation of the MRI image slice. A two-dimensional thickness algorithm was used to combine the segmentation outputs for the 2D representation of images of the end-diastole (ED) and end-systole (ES) cardiac volumes. The thickness images were subsequently used for classification by using a few-shot model with an adaptive subspace classifier. Model performance was verified by applying the model to the 2017 MICCAI Medical Image Computing and Computer-Assisted Intervention dataset. High segmentation performance was achieved as follows: the average Dice coefficients of segmentation were 96.24% (ED) and 89.92% (ES) for the left ventricle (LV); the values for the right ventricle (RV) were 92.90% (ED) and 86.92% (ES). The values for myocardium were 88.90% (ED) and 90.48% (ES). An accuracy score of 92% was achieved in the classification of various cardiomyopathy groups without clinical features. A novel rapid analysis approach was proposed for heart disease diagnosis, especially for cardiomyopathy conditions using cine MRI based on segmented output maps.

## 1. Introduction

Heart diseases, such as coronary, arrhythmia, congenital, and muscle disease (cardiomyopathy) [1], are the leading cause of death worldwide. Cardiomyopathy has attracted considerable research attention because it is typically associated with patients with heart failure [2]. Several cardiomyopathy types, such as dilated cardiomyopathy (DCM), hypertrophic cardiomyopathy (HCM), heart failure due to myocardial infractions (MINFs), and right ventricular abnormalities, have been reported [3]. Early identification of cardiac dysfunction and rapid diagnosis of heart disease can be achieved by evaluating cardiac parameters, such as end-systolic (ES) volume, end-diastolic volume, ejection fraction, and stroke volume, using magnetic resonance imaging (MRI) [4]. Computer-aided automated classification and segmentation solutions have alleviated time-consuming problems in manual diagnostics and interobserver variability [5,6]. Moreover, these problems have spurred the development of the deep neural network (DNN) methods in cardiomyopathy diagnosis.

The output of deep-learning-based cine MRI segmentation can be used as the basis for diagnostically obtaining derived features, such as ventricular volume [7], myocardial mass, and ejection fraction (EF). These derived features are clinical parameters or clinical indices. These values are generally evaluated two times—at the end of the diastolic and systolic phases (ED and ES, respectively). The accuracy of the calculation of clinical parameters depends on the accurate depiction of the contours of the underlying cardiac structures. Isensee et al. [8] used UNet segmentation by combining 2D and 3D modeling to obtain derivative features for diagnosis. Khened et al. (2019) [9] proposed a 2D multiscale fully convolutional network architecture based on residual DenseNet to derive the features of cardiac physiological parameters and handcrafted features. UNet outperforms most other methods; however, the derived features in this technique are obtained by acquiring the slice number and instrument setting, which enhances classification complexity.

The classification of cine MRI based on derived features is highly reliable. Classical machine learning methods, such as random forest [10] and multilayer perceptron (MLP) [8], have been used for disease classification. Khened et al., [11] proposed an ensemble of multiple models for improving classification performance. The small amount of data renders conventional machine learning more suitable than deep learning. Deep learning was also used by Isensee et al. [8] with augmentation and MLP for classification; however, this method included derived information. In deep learning, few-shot learning is highly preferable in areas with the limited data.

The few-shot model was introduced to generalize a new class with a limited number of labeled examples for each class. The meta-learning paradigm was used to achieve accurate DNN models with less training data [12]. Few-shot learning has been implemented through several approaches such as transfer learning [13], metric learning [12,14,15], data augmentation [16], and model optimization [17]. These methods typically work well within the same domain; however, their performance deteriorates in cross-domain applications [18]. Chen et al. [19] proved that feature improvement using a deeper encoder can minimize the variation in scores between methods. The metric learning-based approach exhibits stable performance and can be flexibly used to study the similarities and differences between classes. This phenomenon is more advantageous when the dataset is in the same domain; however, the baseline model [13] is preferable in cross-domain cases.

The adaptive subspace [15] approach is a subspace-based dynamic classifier that has been developed to address data ambiguity with class representations based on subspace mechanisms. This model outperforms previous metric learning methods such as matching networks [12] and prototype networks [14]. The subspace mechanism can be easily customized and can optimize the feature representation in the encoder network and class representation in the subspace classifier, which enables backpropagation. Moreover, episodic learning can be used freely. The use of random permutations for each class that appears for each number of shots and queries can produce many possibilities that can be contained in episodes.

The main contributions of this paper are summarized as follows:

(1) Several lightweight encoders were ensembled using a block-inverted residual network in the UNet architecture for automatic cine MRI segmentation optimization.
(2) We proposed a novel 2D thickness algorithm to decode the segmentation outputs to develop the 2D representation images of the ED and ES cardiac volumes as the input for of cardiac muscle heart disease patient groups classification without using clinical features.
(3) A few-shot model with an adaptive subspace classifier was proposed, and various encoder few-shot models were investigated for deep-learning-based cardiac disease group classification. Unlike the general few-shot learning mechanism, the same classes and domain datasets are provided in the few-shot learning mechanisms in the training, validation, and testing phases.
(4) The ensemble method was used to obtain an even distribution of the class representations from this few-shot mechanism [20]. The source code for the developed models is available at https:/github.com/bowoadi/cine_MRI_segmentation_classification (accessed on 6 July 2022).

## 2. Materials and Methods

### 2.1. Dataset

In this study, the MICCAI 2017 automated cardiac diagnosis challenge (ACDC) dataset was used [21]. The dataset consisted of the cardiac cine image series of one cardiac cycle at various short-axis slice positions, from basal to apical, collected from the examination of 150 patients at the University Hospital of Dijon (France). Two MRI scanners with distinct magnetic strengths (1.5 T–3.0 T) were used to acquire short-axis cine MRI slices. The obtained short-axis MRI cine slices covered the LV and RV from the base (top slice) to the apex (bottom slice), with a slice thickness of 5–8 mm, a gap between slices 5 mm or 10 mm and 1.37–1.68 mm/px as spatial resolution. Each cardiac cycle was covered by 28 to 40 frames in the time dimension. The patients were grouped under five cardiac conditions, namely dilated cardiomyopathy (DCM), hypertrophic cardiomyopathy (HCM), heart failure due to MINFs, abnormal RV (ARV), and healthy or normal patients (NOR); each group contained 30 patients. The ground truth for segmentation had four labels, namely background (0), RV (1), myocardium (2), and left ventricle (3). Information on disease class and ground truth segmentation was provided for 100 patients, whereas the data pertaining to the other 50 patients were used as test data and submitted in the post-competition phase to obtain the test scores.

### 2.2. Proposed Method

**Figure 1:**
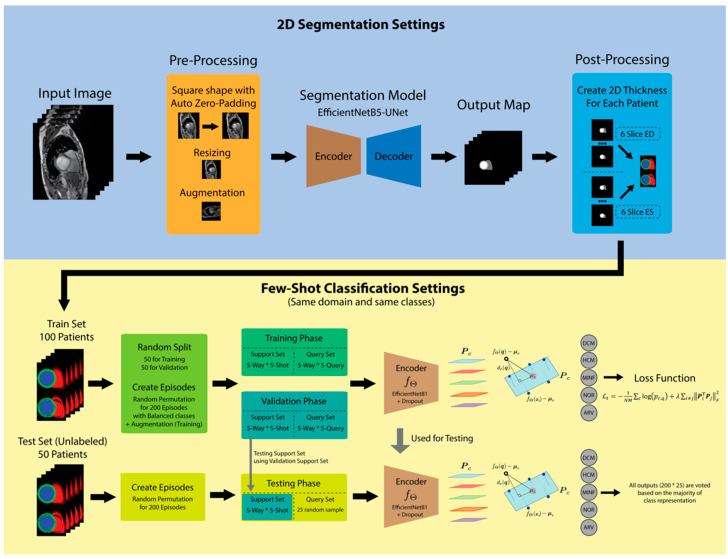
Proposed architecture consisting of 2D segmentation (**top**) and few-shot classification (**bottom**).

The proposed architecture consisted of a 2D segmentation model based on a U-Net-based encoder–decoder structure and a classification model based on few-shot learning (Figure 1). The proposed 2D thickness algorithm connected two tasks to convert the output maps of the 2D segmentation network into a 2D image that represents the thickness of the short-axis slices for each patient. In this experiment, a lightweight model based on an inverted residual network was used [22] for segmentation and classification encoders. EfficientNet is composed of a basic block-inverted residual network used as an encoder and pre-trained weights. This method is intended to strengthen the feature representation for both segmentation and few-shot classification. EfficientNet [23] performance was compared with that of MobileNetV3 [24], which also has an inverted residual network architecture base [25].

### 2.3. Segmentation Model and 2D Thickness Algorithm

The segmentation model was inspired by the lightweight encoder–decoder MobileNetV3-UNet [26]. The lightweight model based on an inverted residual network exhibits high generalization capability, which is faster and lighter, and optimization of the network architecture search, which improves the accuracy of feature representation. UNet is a fast generalized semantic segmentation model, particularly for handling biomedical images [27]. Therefore, to improve performance, the feature representation of the encoder is essential at each stage of the UNet. UNet is used to capture the global and local features through skip connections at each stage. The MobileNetV3-UNet model was modified by replacing the encoder with EfficientNet [23] and subsequently fine-tuning the models from EfficientNetB0 until EfficientNetB5.

**Figure 2:**
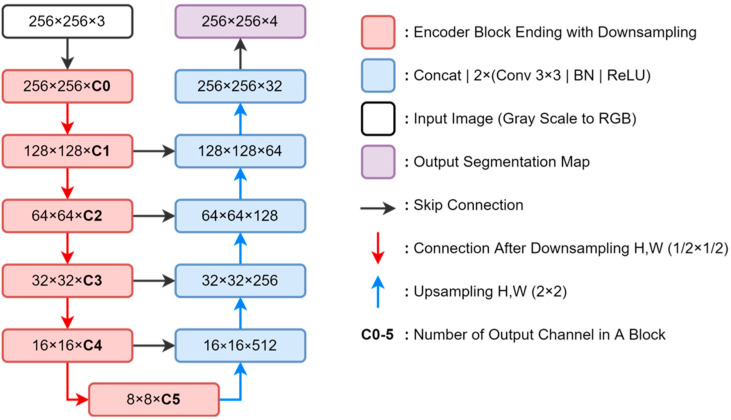
Encoder–decoder UNet suitable for any encoder.

**Figure 3:**
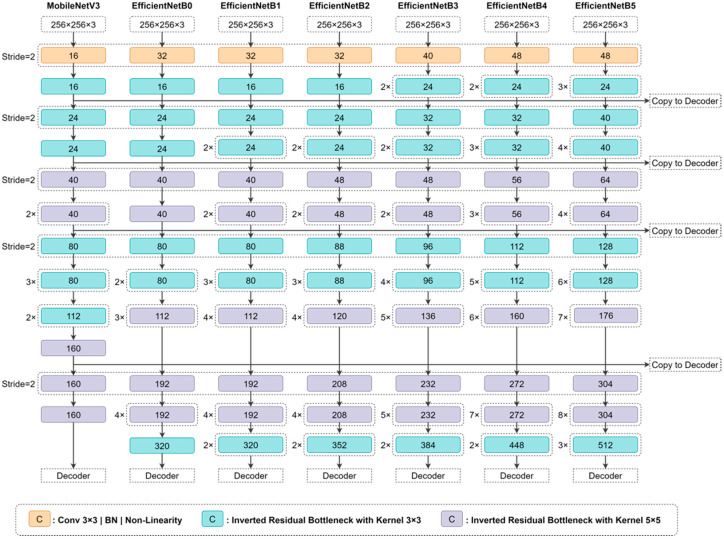
Architecture detail on MobileNetV3 and EfficientNet B0-B5 as the encoder.

Ronneberger et al. first proposed the UNet architecture [27], which consists of a fully convolutional neural network that helps in classifying classes in each pixel with an encoder–decoder pattern. According to Figure 2, the input image is obtained through a series of blocks marked in red, which are contraction pathways or encoders. The encoder can be replaced using state-of-the-art encoder variations, such as EfficientNet [23] and MobileNetV3 [24]. Typically, the downsampling phase is performed from a convolution kernel with two strides, which start directly at the start block; therefore, the red arrow is an illustration of connecting blocks after the downsampling phase. Each downsampling phase with a height *H* and a width *W* of the feature map is halved, and C0 to C5 is the channel size extracted from each block; the amount depends on the variation of the encoder used. Feature maps are sent from the encoder to the decoder through a skip connection; therefore, the resolution of the encoder and decoder should be the same. The lowest resolution block encoding, also called a bottleneck, ends the contraction path and starts the expansion path. Operations opposite to the previous and in reverse order are referred to as expansion paths or decoders, marked in blue. The block decoder consists of convolution 3 × 3 operations followed by batch normalization and ReLU, which are performed two times. The upsampling block gradually increases spatial dimensions (marked with blue arrows) to 2 × 2 of the *H* and W measurements of the feature map. At the final level of the expansion path, the kernel’s projection (convolution block) operation uses 1 × 1 to match the output channel dimensions to the number of segmented classes. Figure 3 displays the interaction of the segmentation network with other networks.

The ensemble method involves combining the pixel-wise classification results from more than a single semantic segmentation model. The first single semantic segmentation model is trained with a composition of input sets of training, validation, and testing. Furthermore, other single semantic segmentation models each receive the same composition of training, validation, and testing input sets as the first single semantic segmentation model. Every semantic segmentation model produces a segmentation output probability. The segmentation output probabilities from these single models are combined and averaged. The softmax function is used to determine the class for each pixel resulting from the average segmentation output probability.

Several preprocessing steps were applied because of variations in the image frame size of each patient. The steps were initiated by applying auto-zero padding to maintain image proportions. In auto-zero padding, zero pixels are added to the vertical or horizontal side in a balanced manner. The obtained square-shaped image was resized to 256 × 256. Finally, the grayscale image was duplicated in three channels. Random augmentation [26] was applied to expand the diversity of images to achieve robustness against complex images. The input size of the segmentation model was 256 × 256 × 3.

The segmentation model produces an output map O with dimensions of 256 × 256 × 4, expressed as H×W×C, where $C=\left[c_{0},\;c_{1},\;c_{2},\;c_{3}\right]$ represents the background, right ventricle (RV), MYO, and left ventricle (LV), respectively. These output maps are used in the 2D thickness algorithm (Algorithm 1) to develop the 2D representation images of the ED and ES cardiac volumes. For all patients, the segmentation output maps were regrouped for the ED and ES frames expressed as $P=\{(p_{0,0},p_{1,0}),\ldots ,\left(p_{0,n-1},p_{1,n-1}\right)\}$, where n is the number of patients. Here, N-slices were considered because of the varying number of short-axis slices in one frame $p_{f}$. Furthermore, N was obtained from the least number of slices in each frame (N=6). Merging was applied by filling the actual pixels of slice k with values from $s_{k}$, where S is a set of N unique values. The merging of the labels of the RV, MYO, and LV forms the red–green–blue (RGB) channel. The background label was not used because the background was automatically created when the three non-background channels were merged. The resulting RGB images I in the ED and ES frames were applied to the center crop, resized to 64 × 64 × 3, and joined vertically for each patient. The final size at post-processing was 128 × 64 × 3.

The post-processing results were used as inputs for the few-shot classification model. The use of the few-shot mechanism in this study differed slightly from its general use. Because in this model, the similarities and differences are learned between the classes, the classes in the support and query samples in the training, validation, and testing phases differed considerably. In this study, the model was trained, validated, and tested by using the same five classes with an episodic mechanism. The prediction output (class representation) for each class in the testing phase was selected through majority voting.

**Algorithm 1** 2D Thickness algorithm
 1: **Function** *thickness(P)* 2:  *S*←[14, 26, 38, 49, 59, 69] 3:  *M*←*empty array with shape of n* × 128 × 64 × 3 4:  for i=0 to n do 5:   for f=0 to 1 do 6:    *I*←*blank image with shape of* 256 × 256 × 3 7:    for k=0 to N do:   8:     $O\leftarrow p_{f,i,k}$ 9:     for c=1 to 3 do:  10:      $\mathrm{if}\;O_{h,w,c}=1\;\mathrm{then}$ 11:       $O_{h,w,c}\leftarrow s_{k}$ 12:      **end if** 13:     **end for** 14:    **end for**  15:    I←I+O 16:    I←crop(I) 17:    I←resize(I,64×64) 18:    $M_{i,f\times 64\ldots ,\left(\left(f+1\right)\times 64\right)-1}\leftarrow I$ 19:   **end for**  20:  **end for** 21:  **return** *M*


### 2.4. Few-Shot Learning Model

The few-shot learning method was used as the classification model to handle limited data. Few-shot terminology is used for training multiple samples in each iteration of the meta-learning mode. In the training process, an episode of ${𝒯}_{i}$ consisted of two types of sets, namely support set $S=\left\{\left(x_{1,1},c_{1,1}\right),\ldots ,\left(x_{N,K},c_{N,K}\right)\right\}$ and query set $Q=\left\{q_{1},\ldots ,q_{N\times M}\right\}$. For each iteration, the number of S and Q was limited based on N-way, which denotes the number of classes, and K-shot or M-query denote the number of samples in each class. The few-shot model has two parts, namely encoder and classifier.

A dynamic classifier based on an adaptive subspace [15] was used in this study. Classification was performed based on the subspace in addition to the shortest distance between the data points and their projection onto the subspace. A set of samples encoded by θ can be expressed as ${\tilde{X}}_{c}=\left[f_{\theta }\left(x_{c,1}\right)-\mu_{c},\;\ldots ,\;f_{\theta }\left(x_{c,K}\right)-\mu_{c}\right]$, where $\mu_{c}=\frac{1}{K}\underset{x_{i}\in X_{c}}{\sum }f_{\theta }\left(x_{i}\right)$. For example, q is the query set, and the subspace classifier calculation is formulated as follows:

(1) $$d_{c}\left(q\right)=-{||\left(I-M_{c}\right)\left(f_{\theta }\left(q\right)-\mu_{c}\right)||}^{2}$$

where $M_{c}=P_{c}P_{c}^{T}$ and $\mu_{c}$ denote the offset between the data point and the subspace, respectively, $P_{c}$ is the truncated matrix of matrix $B_{c}$ with an orthogonal basis for linear subspace spanning $X_{c}=\left\{f_{\theta }\left(x_{i}\right);y_{i}=c\right\}$ (hence, $B_{c}^{T}B_{c}=I$).

The probability of a query entering the class c can be determined using the softmax function is expressed as follows:

(2) $$p_{c,q}=p\left(c|q\right)=\frac{\exp\left(d_{c}\left(q\right)\right)}{\sum_{c^{\prime }}\exp\left(d_{c^{\prime }}\left(q\right)\right)}$$

Backpropagation through singular value decomposition can be used to minimize the negative log from $p_{c,q}$.

The projection metric on Grassmannian geometry was used as a discriminative method to maximize the margin between two subspaces $P_{i}$ and $P_{j}$ during training and is formulated as follows:

(3) $$\delta_{p}^{2}\left(P_{i},P_{j}\right)={||P_{i}P_{i}^{T}-P_{j}P_{j}^{T}||}_{F}^{2}=2n-2{||P_{i}^{T}P_{j}||}_{F}^{2}$$

The projection metric was maximized by minimizing ${||P_{i}^{T}P_{j}||}_{F}^{2}$ followed by formulating a loss function as shown in Equation (4); subsequently, ${ℒ}_{t}$ can be used to update θ.

(4) $${ℒ}_{t}=-\frac{1}{NM}\sum_{c}\log\left(p_{c,q}\right)+\lambda \sum_{i\neq j}{||P_{i}^{T}P_{j}||}_{F}^{2}$$

### 2.5. Segmentation Training and Testing Scenario

Segmentation model training was conducted on an i7 processor with an RTX 2060 SUPER by using a batch size of four for model generalization [28]. A five-fold cross-validation mechanism was used for model training by considering the weights at the best validation for each fold in the first 150 epochs. The data distribution in each fold was separated based on the number of patients, that is, 80 for training and 20 for validation, where each class was set equally. Each patient had a distinct number of short-axis slices; therefore, 1902 images were acquired. The image preprocessed with auto-zero padding and random augmentation was used as the input model with dimensions of 256 × 256 × 3. The grayscale image was copied into three channels to obtain a superior result by using the pre-trained ImageNet model. The Jaccard loss ${ℒ}_{jaccard}$, formulated as Equation (5), was used as the loss function along with the Adam optimizer with a learning rate of 1 × 10^−3^. Finally, the segmentation output was post-processed using the hole-filling method and the largest contour, and an operation was applied to remove the double contour of the RV because it tends to be noisy.

(5) $${ℒ}_{jaccard}=1-\frac{\left|G\cap P\right|}{\left|G\right|+\left|P\right|-\left|G\cap P\right|}$$

where G is the ground truth and P is the output of the segmentation model.

The 2D segmentation model was tested in two stages: a local test conducted with the validation set, and an online test based on the competition leaderboard, conducted with the test set. Both the validation and test sets were preprocessed but not augmented. For each fold on the MobileNetV3-UNet and EfficientNet-UNet models, local tests were conducted using the Dice coefficient evaluation metric. An online test based on the competition leaderboard was performed by submitting the final segmentation results, obtained as the average of the segmentation map output from the five-fold model. During submission, the final segmentation result was returned to its original size first and reassembled into a nii file for each patient. The model was evaluated using several metrics according to the competition leaderboard.

### 2.6. Few-Shot Classification Training and Testing Scenarios

A minimal dataset was used for the few-shot model. Images from the post-processing segmentation model of 100 patients were randomly separated into 50 balanced classes for the training and validation phases. During the testing phase, 50 images were randomly sampled. The settings used were five-way for all phases, five-shot on the support and query sets, and 100 episodes for each epoch. Each episode consisted of 25 support sets and 25 query sets. The training iteration used ten epochs, with a learning rate of 1 × 10^−3^, optimized by Adam, and lambda of 0.03. The few-shot model ran on an i7 processor with RTX 2060 SUPER.

The validation support set was used as the support set test in the testing phase, and 25 queries for each episode were selected randomly from the test data. The majority of the prediction results were voted based on the patient ID. The training–testing process was repeated five times to obtain stable models, and an ensemble was then performed. Experiments were conducted to investigate the effects of using various encoders, augmentation, dropout, and 1–5 shot settings. The best experimental model was considered the final model and submitted to the post-2017-MICCAI-challenge testing phase for diagnosis. The experimental results were evaluated against the validation set based on accuracy.

## 3. Results

### 3.1. Segmentation Results

The proposed model was evaluated in two stages, namely local and online. In the local tests, five-fold cross-validation was used to test MobileNetV3-UNet and EfficientNet-UNet on the validation set using Dice coefficients, as presented in Table 1. Although EfficientNetB3 and EfficientNetB5 obtained the highest scores, the Dice coefficients of MobileNetV3-UNet and EfficientNet-UNet exhibited slightly distinct values. However, the standard deviation of EfficientNetB5-UNet was lower by 0.0024 when compared with that of MobileNetV3-UNet. Because of the insignificant difference, EfficientNetB5 was used for online testing. MobileNetV3 was additionally used, and two ensembles of the models were created. First, Ensemble B0–B5 was generated from the average of the output maps of EfficientNetB0 to EfficientNetB5. Next, the V3B5 ensemble was generated from the average of the output maps of MobileNetV3 and EfficientNetB5. In Table 2, the outcomes of the proposed method and those of several previous methods are displayed in terms of the Dice coefficient evaluation metrics. The test results on the leaderboard revealed that EfficientNetB5-UNet outperformed MobileNetV3-UNet for the LV, RV, and MYO features. Although ensembling did not outperform several methods in the post-2017-MICCAI-challenge testing phase, it improved the average Dice coefficient. Therefore, Ensemble B0–B5 with an average Dice coefficient of 90.8% was used to perform the next task (few-shot classification).

### 3.2. Classification Results

**Figure 4:**
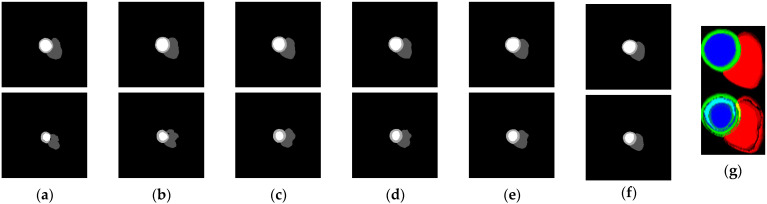
Segmentation output maps (**a**–**f**) of short-axis slices 1–6 of each ED (**top**) and ES (**bottom**) frames processed by the 2D thickness algorithm to produce 2D images (**g**) for few-shot model input.

In the proposed classification method, 2D images are processed as inputs. Figure 4 displays the 2D images obtained by merging the output maps from the segmentation model by using the 2D thickness algorithm. Figure 4g was acquired by combining the first six short-axis slices from the basal slice (Figure 4a–f). In the case of six slices, the combination was from basal to apical. Experiments were conducted to improve the performance of the few-shot model under five scenarios. The first scenario involved determining the effect of using distinct encoders. In the second scenario, we examined the effects of dropout and augmentation. The third step was to locate the best set of shots. The fourth was to observe the effect of various numbers of short-axis slices in the 2D thickness images, and the final step was to determine the best few-shot on the test leaderboard.

Three types of encoders (CNN, MobileNetV3, and EfficientNet) were used in the first scenario. A standard CNN with four convolutional layers (Conv4) was used to determine the feature extraction capability by using a simple convolutional model. MobileNetV3 and EfficientNet are deep and lightweight models based on an inverted residual network with excellent feature representation capabilities [31]. The effects of using pre-trained ImageNet weights and non-pre-trained weights for the deep models were investigated. Table 3 reveals that EfficientNetB1 using the pre-trained ImageNet exhibits a higher average accuracy of 68.68% and lower standard deviation of 0.6883%. The results revealed that deep and lightweight models based on pre-trained models could improve model generalizability, especially in the case of deep models.

In the second scenario, dropout and augmentation were applied. The training results are not presented because the entire training phase exhibited 100% accuracy. However, optimization was applied by adding dropouts and augmentations to the input images. A similar augmentation in segmentation was applied by adding a coarse dropout by removing 4–8 pixels randomly. A dropout of 0.5 was applied at the end of the encoder before entering the classifier. Table 4 lists the effects of augmentation and dropout. Typically, augmentation improves accuracy, whereas dropout degrades accuracy. However, the application of both augmentation and dropout can increase accuracy. EfficientNetB1 with pre-training, dropout, and augmentation resulted in an average accuracy of 78.23%, with the smallest standard deviation.

In the third scenario, the maximum possible number of shots was five because each class had only twenty data points (divided into five for support set and five for query set, with the remaining split between training and validation). However, fewer than five shots were recorded. Table 5 details the optimal number of shots between one and five. A small number of shots can degrade model performance. However, a stable model can be obtained with the five-shot setting.

**Figure 5:**
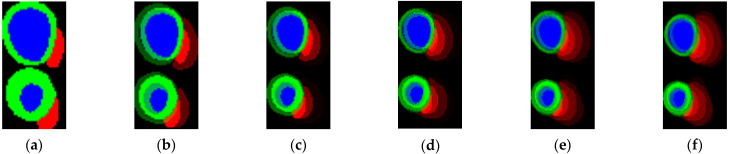
Examples of 2D thickness images for (**a**) 1-slice, (**b**) 2-slice, (**c**) 3-slice, (**d**) 4-slice, (**e**) 5-slice, and (**f**) 6-slice.

In the fourth scenario, the smallest number of short-axis slices from the basal to the apical side in the dataset was six. Therefore, the 2D thickness images were rendered in several slices between one and six. Examples of the results of the 2D thickness algorithm are displayed in Figure 5a 1-slice, (b) two-slice, (c) three-slice, (d) four-slice, (e) five-slice, and (f) six-slice. Using the 2D thickness algorithm, each S pixel value inserted into the slice was 255 for one-slice, (85 for first slice, 170 for second slice) for two-slice, (21, 87, 147) for three-slice, (16, 51, 85, 103) for four-slice, (15, 29, 51, 67, 93) for five-slice, and (14, 26, 38, 49, 59, 69) for six-slice. Different numbers of slices (between one and six) were tested using the best model (EfficientNetB1, pre-trained, dropout, and augmented), as presented in Table 6. The results revealed that higher number of slices improved classification performance, and the performance in the experiment with six-slice was superior to those in the experiments with lesser number of slices because of its rich features.

In the final scenario, because the post-2017-MICCAI-challenge testing phase for diagnosis allowed limited testing, several tests were performed using various accounts. Table 7 lists the test leaderboard scores. Five individual experiments were conducted, and ensemble was used in each of the experiments. In the ensemble model, major voting was used based on the most frequent occurrences of the class. Each model obtained an accuracy score between 78% and 86%, which indicated instability; because the few-shot determined the label by matching the support set, the support set samples were randomly selected. Therefore, an ensemble was created to obtain models with completely random samples. Based on the results, the ensemble model achieved 92% accuracy for diagnosis and could compete with the state-of-the-art models.

## 4. Discussion

Segmentation and classification of cine MRI data are challenging tasks. A U-Net-based encoder–decoder model was used in segmentation to create output maps and classify them into a few-shot model. Several studies have been conducted on cine MRI segmentation (Table 8). The advantages and disadvantages of each method have been listed in the table. In general, UNet is the best framework for this segmentation, and achieves an average Dice score of more than 80. Moreover, the proposed method renders segmentation lighter with UNet-EfficientNetB5 for data per slice, but the segmentation performance does not outperform previous method.

**Figure 6:**
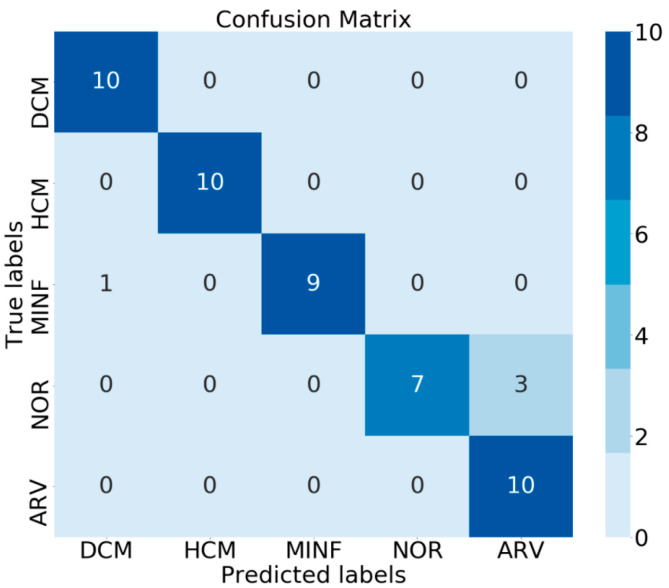
Confusion matrix of ensemble few-shot model on test leaderboard.

For the cine MRI classification presented in Table 8, in most studies non-deep learning methods that include clinical features are used. In the deep learning method, only slices are considered, and model performance does not depend on the parameter settings of the tool and the number of slices obtained. Furthermore, the data can be compared between the number of slices with a small dataset. The few-shot model overcomes the small dataset, especially in image-based tasks. The few-shot learning approach with an adaptive subspace classifier was modified to suit the classification task by training 100 heart disease datasets. The meta-learning paradigm was applied using episodic mechanisms. A task domain with the same class was used for each phase (training, validation, and testing). The encoder plays a critical role in extracting image features. Therefore, using a pre-trained lightweight and deep model based on an inverted residual network improves accuracy. The use of augmentation and dropout increased model robustness. To utilize the few-shot model, an ensemble was generated to obtain an even distribution of class representations from the models. Figure 6 displays the confusion matrix of the results in the test leaderboard. The performance of the model in recognizing the classes ARV-NOR and DCM-MINF was highly biased. Prediction errors still occurred in these classes. Although the accuracy was not the highest on the leaderboard, this model could effectively perform image-based classifications.

**Figure 7:**
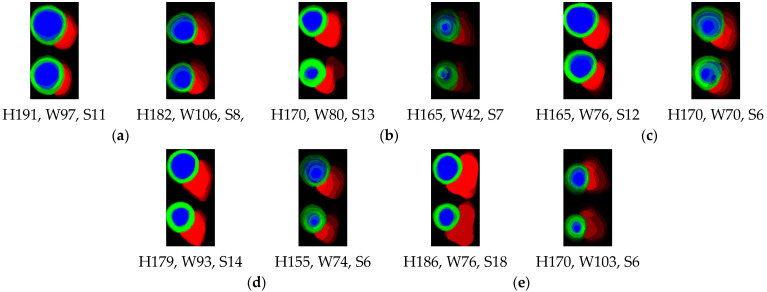
Comparison of results for all cardiac conditions (**a**) DCM, (**b**) HCM, (**c**) MINF, (**d**) normal, and (**e**) ARV, with H: height, W: weight, S: slices.

The application of six short-axis slices plotted for each cardiac condition for various heights/weights (H/W) and the available number of slices in the samples is displayed in Figure 7. The DCM condition with small H/W/S ratios is depicted in Figure 7a. By plotting the ED and ES in alignment, as displayed in Figure 7a, we can observe the cardiac enlargement and ineffective blood pumping in the ES state [33]. The HCM condition is displayed in Figure 7b. The H/W/S ratios differ considerably in this case, and the HCM condition can be observed by plotting the thickness of the heart muscle in comparison with that in other conditions [34]. Figure 7c details the conditions of patients with MINF.

A comparison of ED and ES revealed a left ventricular EF of <40% [35]; furthermore, some myocardial segments with abnormal contractions could be observed. However, this condition is similar to that of DCM, but no enlargement of the heart was observed. Therefore, the padding method is advantageous to remain following its proportions. Normal cardiac conditions are displayed in Figure 7d. Normal cardiac plotting is similar to that of ARV because the RV becomes thicker with layer accumulation, especially when the number of slices is small. Figure 7e displays the ARV condition. The right ventricular volume is higher than usual [36]. Furthermore, the EF of the RV was lower than 40% because it was enlarged despite the ES condition. These results revealed that ED–ES plotting with a 2D thickness algorithm can be used to visualize cardiac conditions with various heights, weights, and slices. Additionally, the proposed method can handle the use of various MRI instruments and settings for classifying cardiac conditions. These results provide numerous opportunities for rapid and straightforward cardiac screening for cardiac diagnosis. In the future, larger sample sizes and classes of cardiac abnormalities should be tested using this approach. Furthermore, 2D thickness and meta-learning experiments for long-axis data represent a novel approach for verifying the detection of various abnormal heart conditions.

## 5. Conclusions

A novel segmentation and classification model was proposed for heart disease diagnosis by using a cine MRI dataset. Encoder–decoder segmentation network EfficientNetB5-UNet was used to perform the semantic segmentation of MRI image slices. A 2D thickness algorithm that combined the segmentation outputs was proposed to develop the 2D representation images of the ED and ES cardiac volumes. The average Dice coefficients of segmentation for the LV were 96.24% (ED) and 89.92% (ES); the values for the RV were 92.90% (ED) and 86.92% (ES), whereas those for MYO were 88.90% (ED) and 90.48% (ES). Subsequently, the 2D thickness images obtained from the model with the best segmentation result were used for the classification to overcome the data shortage problem. The ensemble approach addresses the high uncertainty of prediction results. By using a six-slice 2D thickness image classification, the model could classify heart diseases in the ACDC dataset with an accuracy score of 92%. These results were consistent with those of other studies in which derivatives and other clinical features were used. This image-based classification can be used as a rapid scanning method for diagnosing heart disease.

## Figures and Tables

**Table 1:** Segmentation on the validation set with the Dice coefficient.

Segmentation Model	Fold 1	Fold 2	Fold 3	Fold 4	Fold 5	Average	Standard Deviation
MobileNetV3-UNet	0.891	0.880	0.889	0.896	0.890	0.889	0.0052
EfficientNetB0-UNet	0.884	0.879	0.885	0.888	0.894	0.886	0.0049
EfficientNetB1-UNet	0.897	0.881	0.888	0.890	0.893	0.890	0.0053
EfficientNetB2-UNet	0.890	0.888	0.883	0.887	0.890	0.888	0.0026
EfficientNetB3-UNet	0.898	0.888	0.894	0.891	0.893	0.893	0.0033
EfficientNetB4-UNet	0.889	0.889	0.888	0.893	0.890	0.890	0.0015
EfficientNetB5-UNet	0.896	0.891	0.893	0.894	0.889	0.893	0.0024

**Table 2:** Segmentation on test leaderboard with the Dice coefficient.

State-of-the-Art Methods	LV		RV		MYO	
	ED	ES	ED	ES	ED	ES
Fabian Isensee [8]	0.967	0.935	0.951	0.904	0.904	0.923
Fumin Guo [29]	0.968	0.935	0.955	0.894	0.906	0.923
Georgios Simantris [30]	0.967	0.928	0.936	0.889	0.891	0.904
Mahendra Khened [11]	0.964	0.917	0.935	0.879	0.889	0.898
Ensemble B0-B5 (proposed)	0.963	0.907	0.919	0.865	0.889	0.905
Ensemble V3B5 (proposed)	0.962	0.899	0.929	0.869	0.889	0.905
EfficientNetB5-UNet (proposed)	0.963	0.904	0.929	0.856	0.887	0.903
MobileNetV3-UNet (proposed)	0.960	0.887	0.885	0.858	0.885	0.898

**Table 3:** Accuracy results of application of various encoder few-shot models to the validation set.

Encoder	Experiment 1	Experiment 2	Experiment 3	Experiment 4	Experiment 5	Average	Standard Deviation
Conv4	0.6912	0.6692	0.6916	0.6568	0.6620	0.6742	0.0146
MobileNetV3 without pre-trained	0.4064	0.4092	0.4944	0.4988	0.5568	0.4731	0.0577
MobileNetV3 pre-trained	0.6304	0.5951	0.6140	0.6375	0.7156	0.6385	0.0412
EfficientNetB1 without pre-trained	0.5036	0.3620	0.5532	0.4474	0.6304	0.4993	0.0913
EfficientNetB1 pre-trained	0.6375	0.6916	0.6916	0.7220	0.7320	0.6949	0.0329

**Table 4:** Accuracy results of applying dropout and augmentation on the validation set.

Encoder (Pre-Trained)	Experiment 1	Experiment 2	Experiment 3	Experiment 4	Experiment 5	Average	Standard Deviation
EfficientNetB1	0.6375	0.6916	0.6916	0.7220	0.7320	0.6949	0.0329
EfficientNetB1—dropout	0.5836	0.6952	0.6468	0.5848	0.6712	0.6363	0.0452
EfficientNetB1—augment	0.7368	0.7735	0.6808	0.7423	0.6948	0.7256	0.0363
EfficientNetB1—dropout-augment	0.8083	0.7622	0.7710	0.7900	0.7801	0.7823	0.0159

**Table 5:** Accuracy results of EfficientNetB1 (pre-trained, dropout, augment) with various shots on the validation set.

Number of Shots	Experiment 1	Experiment 2	Experiment 3	Experiment 4	Experiment 5	Average	Standard Deviation
1-shot	0.6360	0.5460	0.5620	0.5639	0.5460	0.5708	0.0348
2-shot	0.7230	0.6780	0.7250	0.7200	0.6919	0.7076	0.0191
3-shot	0.7007	0.7507	0.7120	0.7220	0.7080	0.7187	0.0174
4-shot	0.7880	0.7150	0.7180	0.7410	0.7490	0.7422	0.0263
5-shot	0.8083	0.7622	0.7710	0.7900	0.7801	0.7823	0.0159

**Table 6:** Accuracy results of EfficientNetB1 (pre-trained, dropout, augmented) with various short-axis slices on the validation set.

Number of Slice(s)	Accuracy Score
1	0.6928
2	0.7184
3	0.7272
4	0.7548
5	0.7632
6	0.8083

**Table 7:** Accuracy results of five ensemble few-shot classifications on the test leaderboard.

Method	Score on Leaderboard
Proposed Experiment 1	78
Proposed Experiment 2	80
Proposed Experiment 3	84
Proposed Experiment 4	86
Proposed Experiment 5	86
Proposed Ensemble	92
[11]	100
[8]	92
[32]	92
[10]	86

**Table 8:** Strengths and weaknesses of the proposed method compared to previous methods.

Method	Stage	Strengths	Weaknesses
Proposed	Segmentation	Segmentation becomes lighter with UNet-EfficientNetB5 for data per slice.	The segmentation performance has not outperformed the previous method.
	Classification	Only considers slices and does not depend on the parameter settings of the tool and the number of slices obtained. Can be compared between the number of slices.	This approach is only suitable for morphological problems. Uncertainty is high because depending on the training in each episode, this is handled by the ensemble.
Mahendra Khened [9]	Segmentation	Segmentation using DenseNet which is suitable for limited data.	Segmentation using 2D UNet with dense block doesn’t outperform the ensemble of 2D and 3D DMR-UNet.
	Classification	Classification becomes faster with Random Forest.	The classification most exclusively focus on end-diastole and end-systole features.
Fabian Isensee [8]	Segmentation	Segmentation by combining 2D and 3D UNets slightly improved.	The 3D UNet has large slice gap on the input images, it causes pooling and upscaling operations are carried out only in the short-axis plane. Moreover, the 3D network involves a smaller number of feature maps.
	Classification	Perform ensemble classification by combining MLP and Random Forest.	The ensemble method does not outperform single Random Forest.
Irem Cetin [32]	Segmentation	The training data was manually segmented to produce accurate results.	They computed large number of computations manually. This method tends to overfitting. To prevent from overfitting, they selected the most discriminative features and used SVM for classification.
	Classification	Classification using Support Vector Machines suitable for limited data.	The classification method does not outperform.
Jelmer M Wolterink [10]	Segmentation	The network was designed to contain a number of convolutional layers with increasing levels of dilatation to produce high resolution feature maps.	Convolutional neural network does not exhibit an encoder–decoder architecture.
	Classification	Classification becomes faster with Random Forest.	Classification methods most exclusively focus on end-diastole and end-systole features. It does not outperform other Random Forest.
Fumin Guo [29]	Segmentation	Segmentation by combining UNet and Continuos Max-Flow.	Only for left ventricle. Other methods function for right ventricle and myocardium.
	Classification	N/A	N/A
Georgios Simantris [30]	Segmentation	Networks trains quickly and efficiently without overfitting.	Does not outperform to the state of art featured in the ACDC.
	Classification	N/A	N/A

## Data Availability

The data is available at https://www.creatis.insa-lyon.fr/Challenge/acdc/databases.html (accessed on 1 August 2021).

## References

[1] Kokubo Y., Matsumoto C. (2017). Hypertension Is a Risk Factor for Several Types of Heart Disease: Review of Prospective Studies. Hypertens. Basic Res. Clin. Pract. Adv. Exp. Med. Biol..

[2] Wexler R., Elton T., Pleister A., Feldman D. (2009). Cardiomyopathy: An Overview. Am. Fam. Phys..

[3] Chugh S.S. (2010). Early Identification of Risk Factors for Sudden Cardiac Death. Nat. Rev. Cardiol..

[4] Clough J.R., Oksuz I., Puyol-Antón E., Ruijsink B., King A.P., Schnabel J.A. (2019). Global and Local Interpretability for Cardiac MRI Classification. Lect. Notes Comput. Sci. (Incl. Subser. Lect. Notes Artif. Intell. Lect. Notes Bioinform.).

[5] Ammar A., Bouattane O., Youssfi M. (2021). Automatic Cardiac Cine MRI Segmentation and Heart Disease Classification. Comput. Med. Imaging Graph..

[6] Tong Q., Li C., Si W., Liao X., Tong Y., Yuan Z., Heng P.A. (2019). RIANet: Recurrent Interleaved Attention Network for Cardiac MRI Segmentation. Comput. Biol. Med..

[7] Ma Y., Wang L., Ma Y., Dong M., Du S., Sun X. (2016). An SPCNN-GVF-Based Approach for the Automatic Segmentation of Left Ventricle in Cardiac Cine MR Images. Int. J. Comput. Assist. Radiol. Surg..

[8] Isensee F., Jaeger P.F., Full P.M., Wolf I., Engelhardt S., Maier-Hein K.H. (2018). Automatic Cardiac Disease Assessment on Cine-MRI via Time-Series Segmentation and Domain Specific Features. Lect. Notes Comput. Sci. (Incl. Subser. Lect. Notes Artif. Intell. Lect. Notes Bioinform.).

[9] Khened M., Kollerathu V.A., Krishnamurthi G. (2019). Fully Convolutional Multi-scale Residual DenseNets for Cardiac Segmentation and Automated Cardiac Diagnosis Using Ensemble of Classifiers. Med. Image Anal..

[10] Wolterink J.M., Leiner T., Viergever M.A., Išgum I. (2018). Automatic Segmentation and Disease Classification Using Cardiac Cine MR Images. Lect. Notes Comput. Sci. (Incl. Subser. Lect. Notes Artif. Intell. Lect. Notes Bioinform.).

[11] Khened M., Alex V., Krishnamurthi G. (2018). Densely Connected Fully Convolutional Network for Short-Axis Cardiac Cine MR Image Segmentation and Heart Diagnosis Using Random Forest. Lect. Notes Comput. Sci. (Incl. Subser. Lect. Notes Artif. Intell. Lect. Notes Bioinform.).

[12] Vinyals O., Blundell C., Lillicrap T., Kavukcuoglu K., Wierstra D. (2016). Matching Networks for One Shot Learning. Adv. Neural Inf. Process. Syst..

[13] Hu J., Lu J., Tan Y.-P., Zhou J. (2016). Deep Transfer Metric Learning. IEEE Trans. Image Process..

[14] Jake S., Kevin S., Richard Z. (2017). Prototypical Networks for Few-Shot Learning. Adv. Neural Inf. Process. Syst..

[15] Simon C., Koniusz P., Nock R., Harandi M. Adaptive Subspaces for Few-Shot Learning. Proceedings of the IEEE Conference on Computer Vision and Pattern Recognition.

[16] Wang Y. Low-Shot Learning from Imaginary Data. Proceedings of the IEEE Conference on Computer Vision and Pattern Recognition Workshops.

[17] Finn C., Abbeel P., Levine S. Model-Agnostic Meta-learning for Fast Adaptation of Deep Networks. Proceedings of the 34th International Conference Machinability Learned ICML.

[18] Li X., Yang X., Ma Z., Xue J.-H. (2021). Deep Metric Learning for Few-Shot Image Classification: A Selective Review. arXiv.

[19] Chen W., Wang Y.F., Liu Y., Kira Z., Tech V. (2019). A Closer Look at Few-Shot Classification. international conference Learned Representacion. arXiv.

[20] Dvornik N., Mairal J., Schmid C. (2019). Diversity with Cooperation: Ensemble Methods for Few-Shot Classification. Proc. IEEE Int. Conf. Comput. Vis..

[21] Bernard O., Lalande A., Zotti C., Cervenansky F., Yang X., Heng P.A., Cetin I., Lekadir K., Camara O., Gonzalez Ballester M.A. (2018). Deep Learning Techniques for Automatic MRI Cardiac Multi-structures Segmentation and Diagnosis: Is the Problem Solved?. IEEE Trans. Med. Imaging.

[22] Sandler M., Howard A., Zhu M., Zhmoginov A., Chen L.-C. MobileNetV2: Inverted Residuals and Linear Bottlenecks. Proceedings of the IEEE Conference on Computer Vision and Pattern Recognition Workshops.

[23] Tan M., Le Q.V. EfficientNet: Rethinking Model Scaling for Convolutional Neural Networks. Proceedings of the 36th International Conference Machinability Learned ICML.

[24] Howard A., Wang W., Chu G., Chen L., Chen B., Tan M. Searching for MobileNetV3. Proceedings of the international conference Computability Vision.

[25] Wibowo A., Pratama C., Sahara D.P., Heliani L.S., Rasyid S., Akbar Z., Muttaqy F., Sudrajat A. (2022). Earthquake Early Warning System Using Ncheck and Hard-Shared Orthogonal Multitarget Regression on Deep Learning. IEEE Geosci. Remote Sens. Lett..

[26] Wibowo A., Purnama S.R., Wirawan P.W., Rasyidi H. (2021). Lightweight Encoder-Decoder Model for Automatic Skin Lesion Segmentation. Inform. Med. Unlocked..

[27] Ronneberger O., Fischer P., Brox T. (2015). U-Net: Convolutional Networks for Biomedical Image Segmentation. Lect. Notes Comput. Sci. (Incl. Subser. Lect. Notes Artif. Intell. Lect. Notes Bioinform.).

[28] Keskar N.S., Nocedal J., Tang P.T.P., Mudigere D., Smelyanskiy M. On Large-Batch Training for Deep Learning: Generalization Gap and Sharp Minima. Proceedings of the 5th International Conference Learned Representacion ICLR.

[29] Zhou R., Guo F., Azarpazhooh M.R., Hashemi S., Cheng X., Spence J.D., Ding M., Fenster A. (2021). Deep Learning-Based Measurement of Total Plaque Area in B-Mode Ultrasound Images. IEEE J. Biomed. Heal. Inform..

[30] Simantiris G., Tziritas G. (2020). Cardiac MRI Segmentation with a Dilated CNN Incorporating Domain-Specific Constraints. IEEE J. Sel. Top. Signal Process..

[31] Qin J., Huang Y., Wen W. (2020). Multi-scale Feature Fusion Residual Network for Single Image Super-Resolution. Neurocomputing..

[32] Cetin I., Sanroma G., Petersen S.E., Napel S., Camara O., Ballester M.G., Lekadir K. (2018). A Radiomics Approach to Computer-Aided Diagnosis with Cardiac Cine-MRI. Lect. Notes Comput. Sci. (Incl. Subser. Lect. Notes Artif. Intell. Lect. Notes Bioinform.).

[33] Jefferies J.L., Towbin J.A. (2010). Dilated Cardiomyopathy. Lancet..

[34] Maron B.J., Maron M.S. (2013). Hypertrophic Cardiomyopathy. Lancet.

[35] Thygesen K., Alpert J.S., Jaffe A.S., Chaitman B.R., Bax J.J., Morrow D.A., White H.D., Executive Group on behalf of the Joint European Society of Cardiology (ESC)/American College of Cardiology (ACC)/American Heart Association (AHA)/World Heart Federation (WHF) Task Force for the Universal Definition of Myocardial Infarction (2018). Fourth Universal Definition of Myocardial Infarction (2018). J. Am. Coll. Cardiol..

[36] de Groote P., Millaire A., Foucher-Hossein C., Nugue O., Marchandise X., Ducloux G., Lablanche J.M. (1998). Right Ventricular Ejection Fraction Is an Independent Predictor of Survival in Patients with Moderate Heart Failure. J. Am. Coll. Cardiol..

